# Chemical Characterization and Rumen-Modulating Effects of *Pinus sylvestris* Essential Oil: In Vitro and In Vivo Study

**DOI:** 10.3390/molecules31101769

**Published:** 2026-05-21

**Authors:** Natalia Pachura-Hanusek, Kamila Lewandowska, Anna Burek, Antoni Szumny, Aleksandra Tabiś, Sylwia Banaszkiewicz, Jacek Bania, Robert Kupczyński

**Affiliations:** 1 Department of Environment Hygiene and Animal Welfare, The Faculty of Biology and Animal Science, Wrocław University of Environmental and Life Sciences, Chełmońskiego St. 38c, 50-375 Wrocław, Poland; natalia.pachura@upwr.edu.pl (N.P.-H.); kamila.lewandowska@upwr.edu.pl (K.L.); anna.burek@upwr.edu.pl (A.B.); 2 Department of Biocatalysis and Food Chemistry, Faculty of Biotechnology and Food Science, Wrocław University of Environmental and Life Sciences, Norwida St. 25, 50-375 Wrocław, Poland; antoni.szumny@upwr.edu.pl; 3 Department of Food Hygiene and Consumer Health Protection, Wrocław University of Environmental and Life Sciences, Norwida St. 31, 50-375 Wrocław, Poland; aleksandra.tabis@upwr.edu.pl (A.T.); sylwia.banaszkiewicz@upwr.edu.pl (S.B.); jacek.bania@upwr.edu.pl (J.B.)

**Keywords:** conifer essential oils, GC–MS analysis, rumen fermentation, methane emission, volatile fatty acids

## Abstract

Conifer-derived essential oils have gained attention as versatile natural additives with potential applications in animal production, including influencing microbial processes and supporting environmental sustainability. This study aimed to characterize the chemical composition of selected conifer essential oils (EOs), evaluate their antimicrobial activity against rumen microorganisms in vitro, and assess the effects of *Pinus sylvestris* essential oil on rumen fermentation and methane production under in vitro and in vivo conditions. EOs from *Thuja occidentalis*, *Cupressus sempervirens*, *Juniperus communis*, *Picea mariana*, *Pinus sylvestris*, and *Pinus pinaster* were analyzed by GC–MS, and their inhibitory activity against selected rumen bacteria was determined by MIC and IC_50_ assays. Based on these results, *P. sylvestris* oil was selected for fermentation experiments. Ninety-two volatile compounds were identified, with monoterpenes as the dominant constituents and α-pinene as the major compound in *P. sylvestris* oil. In vitro, *P. sylvestris* oil influenced fermentation in a dose-dependent manner without affecting ruminal pH. In vivo, ruminal pH, ammonia-related parameters, and total VFA concentration were not significantly affected by treatment, whereas several variables showed a significant effect of time. Temporal changes in VFA profiles suggested a transient adaptation of ruminal fermentation. Methane concentration was significantly (*p* < 0.01) reduced by *Pinus sylvestris* essential oil supplementation, with a decrease of approximately 28.7% after 14 days. These findings indicate that *P. sylvestris* EOs may serve as a promising natural modulator of rumen fermentation, although further studies are needed to optimize dosage and confirm long-term effects.

## 1. Introduction

Ruminant livestock production plays a crucial role in global food systems; however, it is also associated with significant environmental challenges, particularly the emission of greenhouse gases such as methane (CH_4_) and nitrogenous compounds including ammonia [1,2]. Methane emissions are especially significant, accounting for approximately 14.5% to as much as 30% of total global anthropogenic CH_4_ emissions [1,3,4]. The process of methanogenesis in the rumen is not only a burden on the environment but also represents a loss of feed energy for the animal, amounting to up to 12% of gross energy intake [3,5,6]. Historically, antibiotic growth promoters, such as ionophores, have been used to improve energy and protein utilization efficiency in the rumen and reduce gas losses [3,7]. However, due to concerns regarding the development of resistant strains and the presence of chemical residues in food, the European Union banned the use of antibiotics in feed in 2006 [8,9,10]. Consequently, strategies aimed at modulating ruminal fermentation have attracted considerable attention as a means to improve feed efficiency and reduce the environmental footprint of livestock production [2,11,12,13]. In response to growing expectations regarding sustainable animal production, intensive research has been initiated on natural additives, such as essential oils (EOs), tannins, and saponins, as well as on synthetic methane inhibitors [14,15,16,17].

In recent years, plant-derived secondary metabolites, including EOs, have emerged as promising natural feed additives for manipulating rumen fermentation [18,19]. EOs are complex mixtures of volatile, lipophilic compounds, primarily terpenes and phenylpropanoids, characterized by antimicrobial, antioxidant, and anti-inflammatory properties. Their mode of action in the rumen is largely attributed to the disruption of microbial cell membranes, inhibition of enzyme systems, and selective suppression of specific microbial groups, including methanogenic archaea and hyper-ammonia-producing bacteria [3,20,21].

A growing body of evidence indicates that EOs can effectively modulate rumen fermentation patterns. Numerous in vitro and in vivo studies have demonstrated their capacity to reduce methane and ammonia production while influencing microbial populations and fermentation end-products [17,21]. In particular, EO blends have been shown to decrease total gas production and methanogenesis while increasing the efficiency of volatile fatty acid production [22,23]. These effects are often dose-dependent and strongly influenced by the chemical composition of the oils, as well as synergistic interactions between individual components. For instance, combinations of cinnamon, peppermint, anise, and clove oils have demonstrated significant reductions in methane emissions while maintaining or even enhancing fermentation efficiency [3,22]. Importantly, EOs may also contribute to improved nitrogen utilization by decreasing ruminal deamination processes, thereby lowering ammonia concentration and enhancing microbial protein synthesis. This dual action—simultaneous mitigation of methane and ammonia emissions—positions EOs as attractive candidates for sustainable livestock production systems [22,24]. Despite these promising findings, the application of EOs is not without limitations. Their efficacy can vary widely depending on dosage, diet composition, and adaptation of rumen microbiota. In some cases, high concentrations may negatively affect feed digestibility due to excessive antimicrobial activity. Therefore, careful selection of compounds and optimization of dosing strategies are critical for achieving desirable outcomes [3,17].

Parallel to the exploration of plant-derived additives, synthetic methane inhibitors have also been developed. One of the most studied compounds is 3-nitrooxypropanol (3-NOP), commercially known as Bovaer^®^ [25]. This compound inhibits methyl-coenzyme M reductase, a key enzyme in methanogenesis [1,5,26]. It has been evaluated in several studies and shown to reduce enteric methane emissions in ruminants under controlled experimental conditions [16,27]. In addition to efficacy, research has also addressed aspects such as effects on rumen fermentation, practical implementation, and administration requirements. These considerations have contributed to increasing interest in natural feed additives, including essential oils, as alternative modulators of rumen fermentation [3,28].

Among the diverse sources of EOs, those derived from coniferous plants (e.g., *Pinus* spp., *Abies* spp., *Thuja* spp.) have recently gained attention as potentially valuable rumen modifiers [29]. Conifer EOs are rich in monoterpenes such as α-pinene, β-pinene, limonene, and bornyl acetate, which exhibit strong antimicrobial and bioactive properties [30,31]. Several studies have indicated that these compounds can selectively inhibit methanogenic archaea and protozoa while modulating bacterial communities involved in fiber degradation and fermentation [14,17]. For example, terpenoid-rich oils from pine and fir have been reported to influence rumen fermentation by reducing methane production and altering volatile fatty acid (VFA) profiles toward increased propionate formation [8,15,32]. Similarly, EOs from *Thuja* species, characterized by high thujone and terpene content, have demonstrated antimicrobial effects against rumen microbes, suggesting their potential in controlling excessive proteolysis and ammonia formation [33,34]. In vitro studies have shown that extracts from the needles of the dense-flowered pine (*Pinus densiflora*) can significantly reduce methane production, with a concentration of approximately 30 mg L^−1^ considered the optimal dose [7]. Similar effects were observed with the use of radiata pine (*Pinus radiata*) bark extract, which reduced the production of ammonium nitrogen (NH_3_-N) and CH_4_ without negatively affecting dry matter digestibility [35]. In vivo studies on goats have shown that the administration of Korean pine (*Pinus koraiensis*) cone oil reduces methane emissions by modifying microflora function, including inhibiting fungal growth and altering pyruvate metabolic pathways [5,29]. Despite promising results, the effectiveness of these natural supplements depends on dosage, chemical composition, and diet type, which requires further verification in various production systems [1,3].

The aim of this study was to quantitatively analyze the composition of conifer essential oils (*Thuja occidentalis*, *Cupressus sempervirens*, *Juniperus communis*, *Picea mariana*, *Pinus sylvestris*, *Pinus pinaster*), determine the minimum inhibitory concentrations (MICs) against selected rumen bacteria in vitro, and then evaluate the effect of pine essential oil on selected rumen fermentation processes and methane production in both in vitro and in vivo experiments. We hypothesize that, due to its composition, pine essential oil exhibits selective antimicrobial activity against selected rumen microorganisms, which may lead to the modulation of fermentation processes and reduce methane production.

## 2. Results

### 2.1. GC-MS Characterization of Essential Oils

The GC–MS analysis allowed for the identification of a total of 92 compounds across the investigated EOs, confirming considerable variability in their qualitative and quantitative composition (Table 1). In general, monoterpenes constituted the predominant group of volatile constituents, although their relative proportions differed markedly depending on the botanical source of the oil. Among the identified compounds, α-pinene was one of the most abundant constituents and represented the dominant component in several oils, particularly in *P. pinaster* (53.25%), *C. sempervirens* (45.06%), and *P. sylvestris* (37.18%). A high proportion of this compound was also observed in *P. mariana* (15.73%), although this oil exhibited a more diversified profile.

The essential oil obtained from *P. mariana* was characterized by the presence of several major constituents, including limonene (21.51%), camphene (18.66%), and α-pinene (15.73%), indicating a more balanced distribution of dominant terpenes. In contrast, *J. communis* showed a distinctly different chemical profile, with terpinene-4-ol (20.06%), γ-terpinene (12.18%), α-terpinene (8.50%), and sabinene (8.27%) among the most important constituents. This composition suggests a relatively greater contribution of oxygenated monoterpenes compared with the other analyzed oils.

A clearly distinct profile was also observed for *T. occidentalis*, in which the main constituents included sabinene (17.09%), fenchone (16.81%), trans-sabinyl acetate (13.54%), β-thujone (11.09%), and bornyl acetate (9.25%). In comparison with the pine and cypress oils, this sample was less strongly dominated by α-pinene and showed a more complex composition with a notable contribution of ketones and ester derivatives. The oil of *C. sempervirens*, apart from its high α-pinene content, also contained considerable amounts of δ-3-carene (25.02%) and β-citronellene (6.06%), whereas *P. sylvestris* was additionally characterized by elevated levels of δ-3-carene (37.52%), borneol (5.33%), and camphene (7.84%). In *P. pinaster*, besides α-pinene, important constituents included δ-3-carene (12.17%), limonene (9.38%), and bornyl acetate (1.33%).

### 2.2. Effects of Essential Oils on Cultures of Rumen Microorganisms

To determine the minimum inhibitory concentrations (MICs) of the tested compounds against beneficial rumen bacterial strains, a series of in vitro culture experiments were conducted. Establishing these MIC values was essential to define the threshold concentrations for safe application in livestock. Notably, at specific optimal concentrations tailored to each strain, a phenomenon of growth stimulation was observed, indicating a dose-dependent response. For *Butyrovibrio fibrisolvens*, the lowest MIC values were observed for *P. pinaster* essential oil (200 ppm) and for *T. occidentalis* and *J. communis* (800 ppm each). Additionally, *T. occidentalis* and *P. sylvestris* exhibited a growth-stimulating effect. With regard to the species *Prevotella albensis*, the highest antimicrobial activity was demonstrated by *P. pinaster* essential oil (Table 2). Moderate activity was observed for *T. occidentalis*, *J. communis*, and *P. sylvestris* (MIC = 800 ppm). No growth-stimulating effect was observed after the application of any of the essential oils. For *Lactobacillus delbrueckii* ssp. lactis, lower sensitivity to the tested essential oils was generally observed. The strongest activity was demonstrated by oils from *P. pinaster* and *C. sempervirens*. *T. occidentalis* oil exhibited a clear growth-stimulating effect on this bacterium. For *Streptococcus bovis*, the lowest MIC values were recorded for oils from *T. occidentalis* and *J. communis*, while the highest were for *P. pinaster*. A growth-stimulating effect was observed exclusively for *T. occidentalis*. In summary, *P. sylvestris* oil was characterized by a unique activity profile, combining moderate inhibitory properties with the ability to stimulate the growth of *Butyrovibrio fibrisolvens*.

### 2.3. Effects of Essential Oils on In Vitro Fermentation

The addition of EO from *P. sylvestris* showed a clear dose-dependent relationship with regard to rumen fermentation kinetics (Table 3). The addition of pine oil did not significantly affect the pH of the incubated rumen contents over a 24-h period. The greatest decrease in pH occurred in the control group. At the higher dose, an increase in proteolytic or deaminative activity in the inoculum was observed, and the NH_3_-N concentration rose to 308.00 mg L^−1^ (compared to 283.73 mg L^−1^ in the control). The lower dose did not cause changes in ammonia concentration. The 25 ppm dose favored maintaining the highest total VFA concentration (1.145 mg mL^−1^), indicating optimization of the fermentation rate without inhibiting microbial activity. The dose caused a statistically insignificant decrease in propionate, with no effect on butyrate content. In contrast, the higher dose also caused an increase in acetate with a marked decrease in propionate and butyrate.

### 2.4. Effects of Pinus Sylvestris Essential Oil Supplementation In Vivo

The effects of *Pinus sylvestris* essential oil supplementation on ruminal fermentation parameters are presented in Table 4. Ruminal pH was not significantly affected by treatment group (*p* < 0.45), and no significant group × time interaction was observed (*p* < 0.13). However, a significant effect of time was found (*p* < 0.01). A slight decrease in pH was observed on day 14 (6.58 in EO vs. 6.70 in CON), with a concurrently higher concentration of total VFAs in the EO group compared to the CON group. Ammonia-related parameters were not significantly affected by treatment group. The group effect was non-significant for NH_3_-N, NH_3_, and NH_4_^+^. In contrast, all three parameters were significantly affected by time (*p* < 0.01), whereas group × time interactions were not significant. In the initial phase of the experiment (days 0 and 3), lower ammonia concentrations were observed in the *P. sylvestris* group. TVFA concentration was not significantly affected by treatment group or by group × time interaction, although a tendency for a time effect was observed (*p* < 0.07). Non-linear changes in the total VFA trend were observed during the experiment. TVFA concentration decreased in the supplemented group on day 7 and increased on day 14. Acetate showed a significant effect of time (*p* < 0.02) and a significant group × time interaction (*p* < 0.01). Propionate and butyrate concentrations were significantly affected by time (*p* < 0.02), whereas the treatment group effect was not significant. The acetate-to-propionate ratio was significantly affected by time (*p* < 0.01), but not by treatment group.

Methane was significantly affected by treatment group (*p* < 0.01) and time (*p* < 0.01), with a tendency toward a group × time interaction (*p* < 0.08). The most significant effect of pine oil supplementation was a marked reduction in methane concentration after 14 days of supplementation. In the experimental group, CH_4_ levels dropped to 15,146.17 ppm, representing a reduction of approximately 28.7% compared to the control group (Table 4).

### 2.5. Microbiome Analysis and Taxonomic Composition

High-throughput sequencing of the V3–V4 region of the 16S rRNA gene generated an average of 272,585 (±50,767 SD) raw read pairs per sample. After quality filtering, 88.77% of reads were retained. Subsequent denoising, merging, and chimera removal using the DADA2 pipeline yielded 177,541 (±33,021 SD) high-quality sequences per sample, corresponding to 65.16% of the initial dataset.

Taxonomic assignment was highly successful across taxonomic ranks, with classification rates reaching 99.77% at the phylum level and 99.47% at the family level.

**Figure 1:**
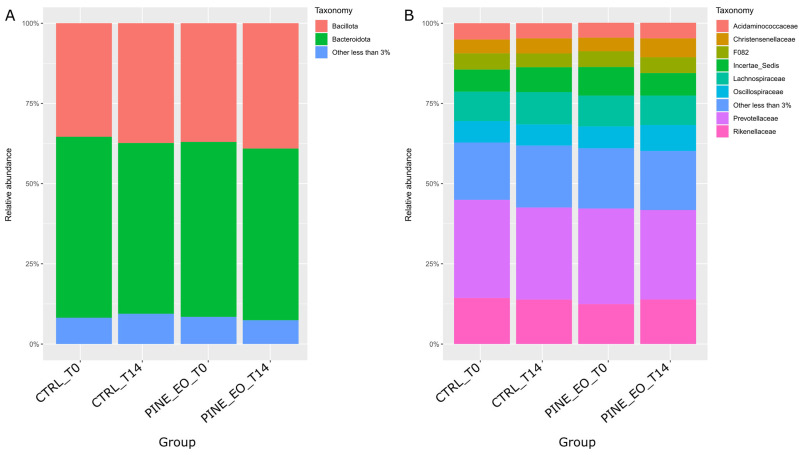
Relative abundance of dominant microbial taxa across experimental groups and timepoints. (**A**) Relative abundance of dominant bacterial and archaeal phyla. (**B**) Relative abundance of dominant bacterial families. Stacked bar plots represent the mean relative abundance (%) in the control and pine essential oil-supplemented groups at baseline (T0) and after 14 days of incubation (T14). Taxa with relative abundance below 3% were grouped as “Other (<3%)”.

The microbial community was dominated by Bacteroidota (54.5 ± 3.8%) and Bacillota (37.1 ± 4.1%), presented in Figure 1A. Other phyla were present at considerably lower abundances. At the family level, Prevotellaceae (29.3 ± 3.7%) and Rikenellaceae (13.7 ± 1.5%) were the most abundant taxa, followed by Lachnospiraceae (9.5 ± 1.6%), Acidaminococcaceae (8.9 ± 0%), Christensenellaceae (7.3 ± 2.7%), and Oscillospiraceae (7.1 ± 1.5%). A substantial fraction of reads (13.7%) remained unclassified (Figure 1B).

Pine essential oil supplementation was associated with limited but statistically significant changes in the relative abundance of specific low-abundance phyla. Chloroflexota showed a modest increase in the supplemented group after 14 days (PINE_EO_T14) compared to both the baseline (CTRL_T0; 0.10 ± 0.03% vs. 0.20 ± 0.07%, *p* = 0.05; Kruskal–Wallis test) and the time-matched control group (CTRL_T14; 0.10 ± 0.06% vs. 0.20 ± 0.07%, *p* = 0.04). In contrast, Cyanobacteriota decreased within the supplemented group over time, from 0.62 ± 0.17% (PINE_EO_T0) to 0.28 ± 0.04% (PINE_EO_T14; *p* = 0.03). At the family level, multiple low-abundance taxa exhibited treatment- or time-dependent differences. These results are provided in Supplementary Table S1.

#### Alpha and Beta Diversity

**Figure 2:**
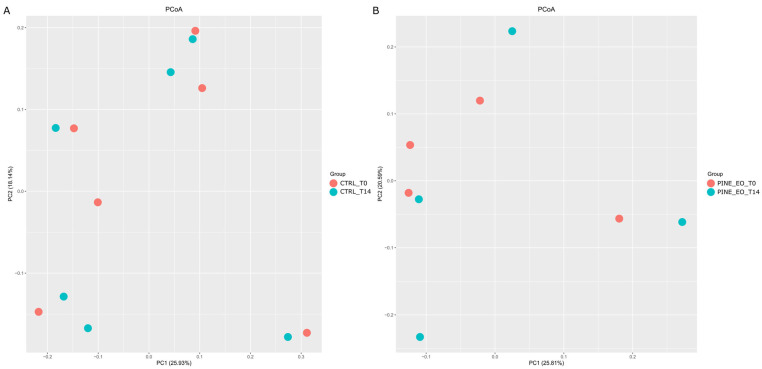
Principal Coordinate Analysis (PCoA) of microbial community structure across experimental groups and timepoints. (**A**) PCoA plot of the control group samples at baseline (CTRL_T0) and after 14 days of incubation (CTRL_T14). (**B**) PCoA plot of the pine essential oil-supplemented group samples at baseline (PINE_EO_T0) and after 14 days of incubation (PINE_EO_T14). Ordination was based on Bray–Curtis dissimilarity matrices calculated from ASV profiles.

Alpha Diversity: Richness increased significantly over time in the control group, as indicated by higher observed ASVs and Chao1 values in CTRL_T14 compared to CTRL_T0 (*p* = 0.04 for both metrics, Welch’s *t*-test; Figure 2). In contrast, no significant changes were detected within the pine essential oil-supplemented group between baseline and day 14 (PINE_EO_T0 vs. PINE_EO_T14; *p* < 0.05). Differences in diversity indices were observed between CTRL_T0 and PINE_EO_T14. Shannon, Simpson, and Inverse Simpson indices were higher following supplementation (*p* = 0.03, *p* = 0.02, and *p* = 0.03, respectively), suggesting increased diversity and evenness in the treated group. No differences were observed between CTRL_T14 and PINE_EO_T14 (*p* < 0.05). Per-sample alpha diversity values are provided in Supplementary Table S2.

Beta Diversity: No significant differences in microbial community composition were observed between CTRL_T0 and CTRL_T14 (Pseudo-F = 1.0568, R^2^ = 0.0956, *p* = 0.38) or between PINE_EO_T0 and PINE_EO_T14 (Pseudo-F = 0.9895, R^2^ = 0.1416, *p* = 0.58). Similarly, no differences were detected between CTRL_T0 and PINE_EO_T14 (Pseudo-F = 1.1043, R^2^ = 0.1213, *p* = 0.32), nor between CTRL_T14 and PINE_EO_T14 (Pseudo-F = 1.1152, R^2^ = 0.1224, *p* = 0.32).

Consistent with these results, PCoA ordination based on Bray–Curtis distances showed no clear separation of samples by treatment or timepoint, with substantial overlap across all groups (PC1 = 20.57–25.93%, PC2 = 17.18–20.59%; Figure 2).

### 2.6. Blood Biochemical Parameters

Supplementation with *P. sylvestris* essential oil had a moderate effect on selected blood biochemical parameters (Table 5). Serum glucose levels were significantly affected by sampling time (*p* < 0.04) and the interaction between treatment and time (*p* < 0.03), whereas the main effect of treatment was not statistically significant (*p* < 0.08). Despite the lack of a significant overall treatment effect, cows receiving *P. sylvestris* EO exhibited higher glucose concentrations on days 7 and 14 compared to the control group (3.53 vs. 3.37 mmol L^−1^ and 3.60 vs. 3.47 mmol L^−1^, respectively). A significant effect of time (*p* < 0.04) and a significant group × time interaction (*p* < 0.03) were observed. BHBA concentration was not significantly affected by treatment group, time, or group × time interaction. However, BHBA values tended to be higher in the supplemented group on day 14 (0.28 vs. 0.22 mmol L^−1^ in CON). NEFA concentration also showed no significant group, time, or interaction effects.

Lipid profile parameters, including total cholesterol, HDL, LDL, and triglycerides, were not significantly affected by treatment group, time, or group × time interaction. Although some numerical differences were observed, particularly for HDL and LDL at later time points, these changes were not statistically significant. Total cholesterol and HDL levels were higher in the group receiving *P. sylvestris* on day 14 (2.52 vs. 2.37 mmol L^−1^ and 1.59 vs. 1.43 mmol L^−1^). Triglyceride concentrations showed a downward trend over time in both groups, with slightly lower values in the supplemented cows. Total protein concentration showed a treatment effect (*p* < 0.05), with higher values observed in the supplemented group. TP levels were consistently higher in the group receiving *P. sylvestris* at all time points. Albumin concentration was not significantly affected by treatment group, but it was significantly affected by time (*p* < 0.04) and by group × time interaction (*p* < 0.01). Liver enzyme activities, including AST, ALT, and GGT, were not significantly affected by treatment group, time, or group × time interaction. Total antioxidant status was also not significantly affected by supplementation. Total antioxidant status (TAS) tended to be higher in the supplemented group on day 14 (1.15 vs. 1.14 mmol L^−1^).

## 3. Discussion

The GC–MS analysis identified a total of 92 volatile compounds across the six investigated conifer essential oils, revealing substantial qualitative and quantitative variability among species. Monoterpenes constituted the dominant class of compounds in all samples; however, their relative proportions differed markedly depending on botanical origin, which is consistent with the known influence of taxonomy as well as environmental and processing factors [36,37].

Among these compounds, α-pinene emerged as a major constituent and, in several cases, the dominant component, particularly in *Pinus pinaster* (53.25%), *Cupressus sempervirens* (45.06%), and *Pinus sylvestris* (37.18%). This predominance aligns with literature reports describing α-pinene as a chemotaxonomic marker of pine and cypress species, typically accounting for 20–60% of total volatiles bao [38,39]. In *P. sylvestris*, the α-pinene/δ-3-carene-rich profile, accompanied by relatively low levels of bornyl acetate, further confirms previously reported compositional patterns [40]. A similar α-pinene-dominated profile was also observed for *P. pinaster*, supporting its classification as a typical pine-type oil.

In contrast, *Picea mariana* exhibited a more balanced chemical profile, characterized by limonene (21.51%), camphene (18.66%), and α-pinene (15.73%), along with notable contributions of bornyl acetate and other oxygenated terpenes. This distribution is consistent with earlier studies indicating a more diversified terpene composition in this species [41]. The essential oil of *C. sempervirens* also followed a typical monoterpene-hydrocarbon chemotype, dominated by α-pinene and δ-3-carene (25.02%). A distinctly different pattern was observed for *Juniperus communis*, where terpinen-4-ol (20.06%), γ-terpinene (12.18%), α-terpinene (8.50%), and sabinene (8.27%) prevailed over α-pinene. This composition reflects a greater contribution of oxygenated monoterpenes and is consistent with previously reported chemotypes, likely associated with plant organ or genetic variability [42,43]. Similarly, *Thuja occidentalis* displayed a clearly divergent profile rich in oxygenated monoterpenes and their derivatives, including fenchone, thujones, sabinene, and ester compounds such as trans-sabinyl acetate. Compared with pine- and cypress-derived oils, this composition indicates greater chemical heterogeneity and a lower dominance of simple monoterpene hydrocarbons [38].

From a functional perspective, the prevalence of compounds such as α-pinene, β-pinene, limonene, sabinene, terpinen-4-ol, fenchone, and thujone derivatives is particularly relevant, as these terpenes are widely associated with antimicrobial activity and the modulation of microbial metabolism [30,44]. Consequently, the pronounced compositional differences observed among the essential oils are likely to be biologically significant and may explain their differential effects on rumen microorganisms, ultimately leading to species-specific fermentation responses. Overall, the results demonstrate that even closely related conifer species can exhibit substantially different chemical profiles, and this diversity should be considered a key determinant of their biological activity and potential application as natural feed additives in ruminant nutrition [45].

One of the major challenges of modern animal production is the mitigation of greenhouse gas emissions, including methane (CH_4_), which represents not only an environmental burden but also a loss of dietary energy. The results of the present study confirm that the essential oil (EO) from *Pinus sylvestris* exhibits substantial potential as a natural modulator of ruminal fermentation, aligning with the ongoing search for alternatives to synthetic methane inhibitors. Choi et al. demonstrated that the cone essential oil of *Pinus koraiensis* effectively reduces methane emissions in goats by modifying ruminal microbial function and inhibiting the growth of ruminal fungi [5,29]. However, direct comparisons should be made with caution due to differences in experimental conditions, including animal species, dosage, and duration of supplementation. This mechanism is attributed to the presence of monoterpenes, such as α-pinene and β-pinene, which predominate in conifer EOs and are known to disrupt the cell membranes of methanogenic archaea [14].

Essential oils from coniferous trees (e.g., pine, spruce, fir) mainly contain terpenes (α-pinene, limonene, terpinen-4-ol) which, in vitro, alter the VFA profile, sometimes increasing propionate and reducing methane while maintaining rumen pH stability [46]. Irrespective of dose and incubation time in vitro, the pH values remained within the physiologic range, as reported by Mensching et al. [47], indicating that the *P. sylvestris* EO did not impair the ruminal buffering capacity and did not pose a risk of metabolic acidosis. Our findings are in agreement with studies in dairy cows, where EO supplementation did not significantly affect ruminal pH despite changes in fermentation patterns [48]. In the in vitro experiment, a dose-dependent response was observed, with the lower dose reducing methane production and the higher dose showing an opposite tendency. Such non-linear responses indicate complex interactions with the rumen microbiota and may reflect differential sensitivity of microbial groups. Importantly, several of the observed changes, including shifts in volatile fatty acids and ammonia-related parameters, were not statistically significant, which limits the strength of biological interpretation. This phenomenon may arise from a disturbance of microbial homeostasis and potential inhibition of hydrogen-competing bacteria (e.g., those utilizing nitrate or sulfate), which in turn favors the methanogenic pathway [49]. The observed reduction in methane emissions at the lower dose of pine EO falls within the range typically reported for botanical additives, which commonly lower CH_4_ emissions by 5–15% without impairing animal performance [50]. This effect is largely ascribed to modulation of the ruminal microbiome, including a decrease in the abundance of methanogenic archaea and altered hydrogen flow [51]. From a practical standpoint, it is relevant that EOs can reduce methane emissions without compromising milk yield [47]. On this basis, the in vivo dose of pine EO in the present study was estimated according to the lower effective dose. Maintenance of stable ruminal pH is crucial, as even small deviations can limit the activity of cellulolytic bacteria and impair fiber utilization [52].

Essential oils may selectively inhibit methanogenic microorganisms while preserving the activity of fermentative bacteria [49]. The present study has certain limitations, as it did not include the identification of specific methanogenic bacterial populations (e.g., *Methanobrevibacter*, *Methanosphaera*). Pine essential oil supplementation had a limited effect on the rumen microbiota. Although significant differences were observed for selected alpha diversity indices and several low-abundance taxa, the overall microbial community structure remained stable. This was supported by the absence of significant differences in beta diversity and the substantial overlap of samples in PCoA ordination. Across all groups, the rumen microbiota was dominated by Bacteroidota and Bacillota (formerly Bacteroidetes and Firmicutes), which typically account for approximately 90% or more of the total taxa in the rumen [53]. While supplementation was associated with statistically significant shifts in several minor phyla, the magnitude of these changes was small in absolute terms. For example, Chloroflexota increased only from approximately 0.1% to 0.2% of the total community, while Cyanobacteriota decreased from approximately 0.6% to 0.3%. Moreover, the majority of significant differences detected at the family level involved taxa present at very low relative abundances, typically below 1% and often below 0.5% of the total community. Therefore, despite statistical significance, the observed effect sizes suggest limited biological relevance at the community level. In rumen 16S datasets, sequences assigned to Cyanobacteriota often represent chloroplast-derived reads from plant material or transient environmental taxa rather than resident bacterial lineages. Minor compositional fluctuations in low-abundance taxa do not necessarily translate into measurable functional consequences due to the high degree of functional redundancy and resilience within the rumen microbial ecosystem [54]. This interpretation is further supported by the stability of beta diversity, indicating that the global microbial community architecture remained largely unaffected by supplementation. Similar patterns, where dietary interventions affect specific minor taxa without altering overall community structure, have been reported previously in rumen microbiome studies [55].

An additional consideration in interpreting the present results is the relatively high proportion of unclassified reads. This is consistent with previous rumen microbiome studies and likely reflects the presence of poorly characterized or uncultured rumen-associated microorganisms, including taxa assigned as F082 and Incertae Sedis. This limitation is largely attributable to the incomplete representation of rumen microbial diversity in current reference databases rather than sequencing quality itself. Although this may reduce taxonomic resolution for specific community members, the dominant community composition and overall comparative patterns between groups remained clearly interpretable.

The increase in propionate proportion alongside reduced methane output indicates a more efficient use of metabolic hydrogen, which is considered one of the primary mechanisms underlying improved energetic efficiency [56]. The observed rise in butyrate production in the present study may be linked to stimulation of cellulolytic bacteria and can positively affect ruminal epithelial health [57]. In terms of nitrogen metabolism, the lack of a persistent decrease in NH_3_-N concentration suggests a limited impact of pine EO on deaminating bacteria, implying that its effect is more focused on methanogenesis than on proteolysis [58]. However, certain EO mixtures have been shown to improve nitrogen utilization by increasing its retention in milk [48]. Compared with synthetic methane inhibitors such as 3-NOP, EOs offer greater consumer acceptance and potentially lower regulatory risk [59].

Recent studies indicate that EO supplementation may influence energy and lipid metabolism in dairy cows by modulating the profile of volatile fatty acids, reducing methane production, and altering the biohydrogenation of fatty acids [1,18,22,60,61]. In addition, the use of EO blends during the transition period of cows may support the metabolic status and immunological functions, thereby improving energy utilization and limiting metabolic disorders [62]. However, the results of the available studies are not consistent, and the effects depend on the type of EO, dose, and interaction with diet and the rumen microbiota. The slightly higher glucose concentration in the supplemented group was associated with the timing of *P. sylvestris* EO administration. The lack of significant changes in NEFA concentration indicates no clear effect on the mobilization of fat reserves, despite minor shifts in energy metabolism. Similar small changes have been reported in studies on EOs, where the impact on energy metabolism was generally limited and inconsistent [15,63]. The increase in glycemia may be related to the modulation of ruminal fermentation (e.g., higher propionate production) and improved utilization of dietary components [64]. The absence of enhanced lipolysis and ketogenesis is supported by other studies as well; EOs rarely affect lipolysis directly and seem to act mainly through changes in ruminal fermentation and microbiota [65].

The most pronounced changes after supplementation with *P. sylvestris* involved lipid-related parameters. An increase in total cholesterol concentration in blood serum and in HDL concentration was observed, with no concurrent rise in LDL. Similar trends of increasing cholesterol have been reported in some studies with EO supplementation, which has been attributed to improved liver function and enhanced lipid transport [15,63,64]. In a few investigations, however, no changes in the lipid profile have been observed [16], confirming the variability of EO effects [66]. Considering the TG concentration, it may be assumed that supplementation did not markedly affect the transport of lipids from the liver to the circulation.

Total protein (TP) concentration was clearly higher in the supplemented group compared with the control, which may indicate increased protein synthesis or a higher proportion of the globulin fraction. Similar effects have been observed in studies where EO supplementation influenced nitrogen utilization and protein metabolism in ruminants, including via modulation of ruminal fermentation and reduced degradation of protein to ammonia [67]. In the present study, no marked increase in ruminal ammonia was detected, especially in the middle of the experiment, which supports the notion that the EO did not substantially enhance proteolysis. The absence of significant changes in albumin concentration suggests that the TP increase arose mainly from alterations in the globulin fraction, possibly reflecting an immunological component of the host response. This is consistent with reports indicating that phytogenic additives, including EOs, can modulate inflammatory and immune parameters [68].

Enzyme activities remained unchanged after supplementation, indicating a lack of hepatocellular damage. This finding agrees with most studies on EOs, which do not report hepatotoxic effects [63]. In contrast, certain phytogenics and EOs have even been shown to exert hepatoprotective actions [64,68]. Some works suggest a potential hepatoprotective effect of EOs stemming from their antioxidant and anti-inflammatory properties [64]. This is further supported by other investigations reporting hepatoprotective activity of phytogenics based on eugenol, cinnamaldehyde, or oleuropein [69,70]. In the present study, liver enzyme activities (AST, ALT, and GGT) remained within normal ranges and were not significantly affected by supplementation, indicating no evidence of hepatocellular damage under the conditions of the present experiment. Although slight numerical differences were observed, they were not statistically significant and should not be overinterpreted. A small increase in total antioxidant status was observed in the supplemented group; however, this change was not statistically significant. Therefore, while it may suggest a tendency toward enhanced antioxidant capacity, firm conclusions cannot be drawn. This is consistent with other reports showing that EOs containing terpenes and phenols can enhance the antioxidant capacity of the organism and mitigate oxidative stress in ruminants [68,71]. In additional studies, phytogenic compounds have been reported to increase the activity of antioxidant enzymes such as glutathione S-transferase (GST) and glutathione peroxidase [68,69]. These findings suggest an improved redox balance, crucial for maintaining homeostasis, possibly due to reduced consumption or limited degradation of endogenous antioxidants under the protective action of such supplements.

α-Pinene, the dominant component in *Pinus sylvestris* essential oil and a major monoterpene present in coniferous EOs, has been shown to exhibit antioxidant activity by scavenging reactive oxygen species and enhancing endogenous antioxidant defenses, including enzymatic systems involved in redox balance [72]. In a model of paracetamol-induced liver injury, α-pinene was found to reduce AST and ALT activities and improve antioxidant indices (GSH, SOD, CAT), while simultaneously decreasing lipid peroxidation, indicating its ability to protect hepatocytes from oxidative stress induced by toxins [72,73]. α-Pinene exerts a protective effect on the liver by increasing the activity of enzymes such as catalase, SOD, and the glutathione system, as well as by inhibiting the activation of the NF-κB pathway, which plays a key role in regulating the expression of pro-inflammatory cytokines [73,74]. Meta-analyses on EO supplementation in ruminants have shown that their addition can increase the activity of endogenous antioxidant enzymes (e.g., catalase and SOD) and total antioxidant potential while simultaneously reducing markers of oxidative stress, indicating a favorable influence on the animals’ redox status. Such supplements may also indirectly modulate protein metabolism and the immune status [65]. In the study of Leal et al., the use of an EO blend with capsaicin in lactating cows improved antioxidant and immunological parameters, further confirming their anti-inflammatory and protective actions [68]. Overall, the present findings indicate that *Pinus sylvestris* essential oil may influence rumen fermentation and methane production under certain conditions; however, the effects are moderate, time-dependent, and not always consistent across parameters. The observed responses suggest a transient adaptation of the rumen ecosystem rather than a strong direct effect. Several interpretations remain speculative due to the lack of targeted mechanistic analyses. Future studies should focus on dose optimization, long-term adaptation effects, and direct investigation of microbial pathways involved in methanogenesis and host metabolism.

## 4. Materials and Methods

### 4.1. Chromatographic Analysis of Essential Oils

The EOs examined in this study were obtained from commercial suppliers with established market credibility, namely Herbiness Sp. z o.o. (Chomiec, Poland) and Avicenna Oil (Wrocław, Poland). The oils were selected based on their widespread availability and previously reported biological activities described in the literature. To ensure traceability, batch numbers of all analyzed oils were recorded as follows: *Thuja occidentalis* (batch no. NEW0121), *Juniperus communis* (batch no. ROZ1116), *Pinus sylvestris* (batch no. 160015), *Pinus pinaster* (batch no. ROZ0321), *Cupressus sempervirens* (batch no. B112/24), and *Picea mariana* (batch no. NEW0120). All experiments were conducted using oils originating from single batches. Due to equipment availability and analytical requirements, different GC–MS systems were used in this study. One instrument was reserved exclusively for volatile organic compound analysis, including essential oils, SPME samples, and trace-level VOC determinations, and therefore was not used for injections of matrices containing non-volatile residues, such as those encountered in VFA analysis. This separation of analytical workflows helps maintain the detector and ion source in optimal condition, ensuring low limits of detection (LOD) and high sensitivity for VOC analyses performed in this and other projects.

Prior to chromatographic analysis, each sample was prepared by diluting 20 µL of essential oil with 980 µL of dichloromethane (GC-MS grade; Sigma-Aldrich, Darmstadt, Germany). The resulting solutions were then transferred to chromatographic vials. Chemical profiling was carried out using gas chromatography coupled with mass spectrometry (GC-MS) with the use of Shimadzu GC-MS QP 2020 (Shimadzu, Kyoto, Japan) and Bruker Scion 8900 GC-MS (Bruker Daltonics, Billerica, MA, USA) systems. Separation of volatile constituents was achieved on a Zebron ZB-5 MSi capillary column (30 m × 0.25 mm × 0.25 µm; Phenomenex, Torrance, CA, USA).

The instrumental parameters were as follows: helium was used as the carrier gas at a constant flow rate of 1.08 mL min^−1^, and the split ratio was set to 1:49. Mass spectra were recorded over the range of 50–350 *m*/*z* at a scan speed of 3 scans s^−1^. The oven temperature was initially maintained at 70 °C and then raised to 200 °C at a rate of 5 °C min^−1^, followed by a further increase to 280 °C at 20 °C min^−1^. The final temperature was held for 3 min, giving a total analysis time of 34.0 min. A 1 µL portion of each prepared solution was injected at 260 °C.

Volatile compound identification was performed using a multi-criteria approach. Mass spectra recorded for individual chromatographic peaks were matched against the NIST 23 (National Institute of Standards and Technology) and FFNSC (Flavors and Fragrances of Natural and Synthetic Compounds) spectral libraries. Data processing and spectral comparison were conducted using AMDIS software (v. 2.73) and GCMSsolution (v. 4.20; Shimadzu, Kyoto, Japan). In addition, linear retention indices (LRIs) were calculated with a retention index calculator and subsequently checked against reference values available in the NIST 23 and FFNSC databases.

### 4.2. Cultures of Rumen Microorganisms

The influence of EOs on the growth of ruminal bacterial strains was evaluated using liquid Schaedler’s broth inoculated with a single colony recovered from solid culture medium. The bacterial strains used in this study were the same as those described in our previous work [18]. Following 48 h of anaerobic incubation at 39 °C, the obtained starter cultures were introduced into a series of twofold EO dilutions. The inoculum density was adjusted to correspond to 0.5 McFarland standard, according to CLSI recommendations [75].

Essential oils were initially prepared in DMSO at a 1:1 ratio and aseptically added to the culture medium after sterilization in order to obtain final concentrations between 25 and 6400 ppm. The assays were carried out in 96-well microplates. Plates inoculated with *Lactobacillus delbrueckii lactis* and *Streptococcus bovis* were incubated anaerobically for 24 h at 39 °C, whereas those containing *Prevotella albensis* and *Butyrivibrio fibrisolvens* were incubated for 48 h at the same temperature. Anaerobic conditions were maintained using a GasPak EZ anaerobe pouch system (BD).

Bacterial growth was monitored spectrophotometrically by measuring optical density at 650 nm with a Spark microplate reader (Tecan, Männedorf, Switzerland). All microbiological procedures were conducted in a Coy Anaerobic Chamber under strictly anaerobic conditions, with oxygen levels maintained below 0.5%.

Based on these measurements, the MIC (minimum inhibitory concentration) and IC_50_ (half-maximal inhibitory concentration) values were established. The percentage inhibition for each EO concentration was calculated relative to the untreated growth control. MIC was defined as the lowest EO concentration causing 90% inhibition of bacterial growth, while IC_50_ was taken as the concentration at which the measured optical density was reduced to 50% of the control value.

### 4.3. Animal Design and Rumen Fluid and Blood Collection

All procedures involving animal cannulation were conducted with the approval of the Local Ethics Committee on Animal Experiments (Protocol No. 053/2019). Prior to the experiment in which the essential oil was used, rumen contents were collected for in vitro testing. Rumen fluid was collected from non-lactating Polish Holstein–Friesian cows fitted with ruminal cannulas, with an average body weight of 600 ± 30 kg. The animals were fed a daily ration consisting of grass silage, hay, rapeseed meal, and a mineral–vitamin supplement, while water was provided ad libitum. Rumen contents were sampled through the fistulas of cannulated cows approximately 3 h after the morning feeding, and about 3 L of fluid was collected each time. Immediately after collection, the rumen fluid was placed in insulated thermoses pre-warmed to maintain a temperature of 39 °C. Prior to further experimental use, the material was filtered through three layers of cheesecloth.

The study on the use of *P. sylvestris* essential oil involved four non-lactating cows fitted with cannulas, in a crossover design, with a single type of supplementation (EO). The cows were housed in divided cubicles with free access to feed and water throughout the day. The total duration of supplement administration was 14 days, followed by a 14-day break before the next treatment cycle. The animals received 2.5 mL/head/day of *P. sylvestris* essential oil via a rumen cannula. The amount of the supplement was determined based on in vitro studies, assuming that a lower dose was more beneficial for TVFA production. It was assumed that the rumen fluid content of a cow is actually approximately 125 L, and the dose of essential oil in the in vivo experiment was estimated based on this.

Rumen contents were collected on days 0, 3, 7, and 14. Methane analyses were performed at similar time points. Blood samples for testing were collected at the start of the experiment and on days 7 and 14. Blood samples were collected into tubes by venipuncture (arteria coccygea mediana). Blood samples were collected in sterile serum tubes (Sarstedt, Nümbrecht, Germany). For serum preparation, samples were centrifuged at 3000× *g* for 10 min at room temperature within two hours of collection, then stored at −20 °C until analysis. Biochemical analyses were performed using a Pentra 400 analyzer (HORIBA ABX Diagnostics, Montpellier, France). Non-esterified fatty acids (NEFA) and beta-hyroxybutyrate acid were determined enzymatically with Randox reagents (Crumlin, Dublin, Ireland). Glucose levels were measured first using the glucose oxidase method (Horiba ABX, Montpellier, France). Subsequently, non-esterified fatty acids (NEFA) were determined enzymatically with Randox reagents (Crumlin, Dublin, Ireland), and triglycerides (TG) and total cholesterol were measured using enzymatic assays with Horiba ABX reagents (Montpellier, France). High-density (HDL) and low-density (LDL), total protein (TP) and albumin (Alb.) lipoprotein cholesterol were quantified by colorimetric assays (Horiba ABX, Montpellier, France). Liver enzyme activities, including aspartate aminotransferase (AST) and gamma-glutamyl transferase (GGT), were assessed using kinetic methods with Horiba ABX reagents.

Total antioxidant status (TAS) in serum was assessed using a colorimetric method based on ABTS (2,2′-azino-bis[3-ethylbenzothiazoline-6-sulfonic acid]) in the presence of peroxidase. Glutathione reductase (GR) activity in whole blood was measured enzymatically; this enzyme catalyzes the reduction of oxidized glutathione (GSSG) using NADPH, which is oxidized to NADP+, with the absorbance change monitored at 340 nm. The analyses were conducted with the use of a Synergy multi-mode microplate reader equipped for fluorescence, luminescence, and absorbance measurements (BioTek Instruments, Winooski, VT, USA).

### 4.4. In Vitro Fermentation and Experimental Design

In vitro fermentation was carried out using the Ankom RF gas production system (ANKOM Technology, Macedon, NY, USA). For each incubation, rumen fluid was first homogenized and then mixed with buffer solution preheated to 39 °C at a ratio of 1:4, corresponding to 75 mL of rumen fluid and 300 mL of buffer according [76]. The buffer solution consisted of two components. Solution A (per 1 L distilled water) contained KH_2_PO_4_ (10 g), MgSO_4_ 7H_2_O (0.5 g), NaCl (0.5 g), CaCl_2_ 2H_2_O (0.1 g), and urea (0.5 g), while solution B contained Na_2_CO_3_ (15 g) and Na_2_S 9H_2_O (1 g). The final pH of the buffer was adjusted to 6.8. The prepared inoculum was subsequently dispensed into 500 mL glass fermentation bottles that had been pre-warmed prior to use. Each bottle contained Ankom bags filled with 1 g of feed material, and in the experimental treatments additional bags supplemented with selected essential oils were introduced. Before incubation, the inoculum was saturated with carbon dioxide and the bottles were placed in a shaking water bath maintained at 39 °C. Throughout the procedure, fermentation was performed under anaerobic conditions and constant temperature to ensure stable microbial activity.

The essential oil used in the incubation experiment was selected based on the results of the preceding in vitro analyses of rumen microbial cultures described in Section 2.2. The experimental design included a control group without essential oil addition (CON) and treatment groups supplemented with *P. sylvestris* essential oil at two volumes, namely 25 and 50 ppm (added to the designated Ankom bags). These doses were chosen for further evaluation in the in vitro fermentation system.

### 4.5. Sample Collection and Ammonia Analysis (In Vitro and In Vivo)

After 4 h and 24 h of incubation, fermentation fluid samples were collected and transferred into 15 mL Falcon tubes in 10 mL portions. To stop further fermentation processes and protect volatile acids from degradation, each sample was immediately stabilized with 0.5 mL of concentrated sulfuric acid (H_2_SO_4_). Prior to analysis, 1 mL of the acidified material was centrifuged at 14,500× *g* for 4 min in order to separate suspended particles. The obtained supernatant was then diluted with deionized water at a ratio of 1:9 to ensure that the ammonia concentration remained within the linear measurement range of the instrument.

Ammonia determination was carried out using a portable colorimeter (Hanna Instruments, Smithfield, RI, USA; model HI-97733) with dedicated reagents, following the manufacturer’s instructions based on the Nessler method ASTM D1426-92 [77]. The final ammonia concentration was calculated automatically by the instrument using the internal calibration settings and expressed in mg L^−1^.

### 4.6. Methane Determination (In Vitro and In Vivo)

Methane (CH_4_) concentrations were determined using gas chromatography. Analyses were performed with a Shimadzu GC-2030 gas chromatograph (Shimadzu Corporation, Kyoto, Japan) equipped with a flame ionization detector (FID) and an SH-Q-BOND capillary column (30 m × 0.32 mm × 10 μm). The injector temperature was set at 240 °C and operated in split mode (10:1). Helium was used as the carrier gas at a linear velocity of 35.0 cm s^−1^. The oven temperature program was as follows: initial temperature of 40 °C (held for 2.5 min), increased at 35 °C min^−1^ to 200 °C, and held for 2.0 min (total run time: 9.07 min). The FID temperature was maintained at 240 °C, with gas flow rates of 32.0 mL min^−1^ for hydrogen, 200.0 mL min^−1^ for air, and 24.0 mL·min^−1^ for helium as makeup gas.

In vitro methane production was evaluated during fermentation experiments. Gas samples were collected from the headspace of incubation vessels at 4 and 24 h using gas-tight Hamilton syringes (600 μL) and immediately injected into the GC system for analysis.

In vivo methane samples were collected into 1 L Tedlar^®^ gas sampling bags (Sigma-Aldrich, St. Louis, MO, USA) connected to a sterile sampling system. Gas was withdrawn using a calibrated portable GilAir-3 EX/ATEX pump (Sensidyne, St. Petersburg, FL, USA) at a constant flow rate of 2 L min^−1^. Before sampling, the cannula opening was carefully sealed to prevent contact with atmospheric air and to maintain anaerobic conditions in the rumen headspace. The sampling line was flushed before each collection to avoid cross-contamination between samples. Gas was collected into pre-evacuated Tedlar^®^ bags until the target volume of 1 L was reached. The bags were immediately sealed, labeled, and transported to the laboratory under ambient conditions, protected from direct sunlight, and analyzed without delay. For analysis, gas samples were introduced directly from the Tedlar^®^ bags via a gas-tight connection to the GC sampling loop, ensuring minimal sample loss and avoiding contamination. Measurements were performed on days 0, 3, 7, and 14 under standardized conditions.

### 4.7. Volatile Fatty Acid Analysis

For the determination of volatile fatty acids (VFAs), 1 mL aliquots collected from the incubation experiment were transferred into 2 mL Eppendorf tubes. Subsequently, heptanoic acid (C7), used as an internal standard, was added to each sample in an amount corresponding to 0.5 mg. Next, 0.8 mL of diethyl ether was introduced, and the samples were vigorously mixed on a vortex mixer for 30 s, followed by centrifugation for 4 min at 14,000× *g* using a benchtop centrifuge. After phase separation, the organic layer was collected and purified by filtration through a celite layer.

The remaining aqueous phase was subjected to a second extraction with 0.8 mL of diethyl ether. After repeated vortex mixing for 30 s and centrifugation for 4 min at 14,000× *g*, the obtained extract was filtered in the same manner. The ether fractions from both extraction steps were then combined, transferred to chromatographic vials, and analyzed by gas chromatography–mass spectrometry (GC-MS).

VFA profiling was performed using the same GC-MS instrument as that applied for essential oil analysis (Shimadzu GCMS QP 2020, Shimadzu, Kyoto, Japan). Separation was achieved on a Zebron ZB-FAME capillary column (60 m × 0.25 mm × 0.20 μm; Phenomenex, Torrance, CA, USA). The oven temperature program began at 80 °C and was held for 1 min, then increased to 200 °C at a rate of 7 °C min−1, with a final hold of 2 min. Mass spectra were recorded over a range of 42–300 *m*/*z* at a scan rate of 1 scan s^−1^. Helium was used as the carrier gas at a flow rate of 1.80 mL min^−1^. The injector temperature was maintained at 260 °C, and the split ratio was set to 1:49. Quantification of individual VFAs was based on calibration curves prepared using authentic reference standards.

### 4.8. Microbial Community Analysis

Four experimental groups were defined to evaluate the effects of pine essential oil supplementation in dairy cows over a 14-day in vivo experiment: CTRL_T0 and CTRL_T14 representing the control cows at baseline and after 14 days, and PINE_EO_T0 and PINE_EO_T14 representing the treated cows before and after supplementation, respectively. The control group included six biological replicates per timepoint, whereas the treatment groups comprised four biological replicates each.

#### 4.8.1. DNA Extraction and Purification

Rumen fluid samples were collected and filtered through sterile gauze to remove large feed particles, then immediately stored at −80 °C until further processing. Genomic DNA was extracted using the MasterPure DNA Purification Kit (LGC Genomics, Teddington, UK) according to the manufacturer’s protocol. An additional clean-up step was performed using the GeneMATRIX Stool DNA Purification Kit (EURx, Gdańsk, Poland) to improve DNA purity.

#### 4.8.2. Library Preparation and Sequencing

Amplification of the V3–V4 region of the bacterial 16S rRNA gene was performed using a two-step PCR approach with primers 341F (5′-CCTACGGGNGGCWGCAG-3′) and 785R (5′-GACTACHVGGGTATCTAATCC-3′). The first PCR generated target amplicons, while the second step incorporated dual indices and Illumina-compatible sequencing adapters. Following each PCR step, amplicons were purified using magnetic bead-based clean-up. DNA concentration was quantified fluorometrically using PicoGreen (Thermo Fisher Scientific, Waltham, MA, USA). Paired-end sequencing was carried out on the Aviti platform (Element Biosciences, San Diego, CA, USA) at Genomed S.A. (Warsaw, Poland).

#### 4.8.3. Bioinformatics Processing

Raw reads were demultiplexed and converted to FASTQ format using vendor-provided software. Downstream processing was conducted in QIIME2 (version 2024.5) [78], using the SILVA 138.2 reference database [79]. Adapter and primer sequences were removed using Cutadapt (version 4.7) [80], followed by quality filtering based on a minimum read length of 30 bp. Denoising, paired-end read merging, and chimera removal were performed using the DADA2 plugin [81], resulting in Amplicon Sequence Variants (ASVs). Taxonomic assignment was performed using a hybrid approach against the SILVA database. Initial classification was conducted using VSEARCH [82] based on sequence similarity (minimum identity 50% and coverage 80%), followed by refinement of unclassified sequences using a Naive Bayes classifier with a confidence threshold of 0.7.

Alpha diversity was assessed using Observed ASVs, Chao1, Shannon, Simpson, and Inverse Simpson indices to capture both richness and evenness of microbial communities. Beta diversity was evaluated using Bray–Curtis dissimilarity matrices and visualised through Principal Coordinate Analysis (PCoA).

### 4.9. Statistical Analysis

Statistical analyses were performed using the Statistica software package (ver. 13, Software Inc., New York, NY, USA). Before performing the actual analyses, the assumptions of the statistical model were verified. The normality of the distribution of variables and residuals was assessed using the Shapiro–Wilk test. For in vitro data, where observations were independent, a two-way ANOVA was applied, followed by Tukey’s post hoc test when appropriate. To account for the repeated-measures design (sampling of the same animals over multiple time points), in vivo data (blood biochemical parameters and rumen fermentation indices) were analyzed using a linear mixed-effects model. In this model, supplementation (Group: Control vs. *Pinus sylvestris* EO) and sampling day (Time: 0, 7, and 14 days) were included as fixed effects, along with their interaction (Group × Time). The individual animal was included as a random effect to account for inter-individual variability and the non-independence of repeated observations. Results are presented as means ± standard error of the mean (SEM). The significance of fixed effects and their interaction is reported as follows: PG for the effect of supplementation (Group), PT for the effect of time (Time), and PG × T for the interaction between Group and Time. Where significant effects were observed, post hoc comparisons were performed using Tukey’s HSD test. In the case of significant effects, the results were presented as means along with the standard error of the mean (SEM). Differences were considered statistically significant at *p* ≤ 0.05, while values of 0.05 < *p* ≤ 0.10 were interpreted as indicating a statistical trend. Differences in microbial community structure between groups were tested using permutational multivariate analysis of variance (PERMANOVA) based on Bray–Curtis distances.

## 5. Conclusions

Supplementation with *Pinus sylvestris* essential oil demonstrated potential as a natural modulator of ruminal fermentation, influencing key metabolic processes under both in vitro and in vivo conditions. The oil did not disrupt ruminal pH stability, indicating its safety with respect to maintaining an appropriate fermentative environment. The in vitro effects of the oil were clearly dose-dependent. Under in vivo conditions, pine oil supplementation led to a substantial reduction in methane emissions (~28%), accompanied by an increase in total volatile fatty acid (VFA). Changes in TVFA and propionate concentrations showed numerical differences between groups, suggesting a shift in fermentation pattern. The absence of a persistent effect on ammoniacal nitrogen concentration indicates ruminal microbiome adaptation and a limited long-term impact on protein metabolism. In dairy cows, supplementation induced moderate yet consistent changes in selected metabolic and lipid parameters. No changes in hepatic enzyme activity (AST, ALT, GGT) were observed during the study period. A slight increase in total antioxidant status (TAS) suggests moderate antioxidant properties of the oil. Overall, *P. sylvestris* essential oil showed potential to modulate ruminal fermentation and methane production. However, the short duration of the study and the limited set of endpoints do not allow definitive conclusions regarding long-term metabolic or physiological effects. Further research is required to evaluate its impact on animal performance, ruminal microbiome function, and long-term metabolic responses.

## Figures and Tables

**Table 1:** Chemical composition of selected essential oils determined by GC–MS, expressed as percentage of total peak area.

No	Peak Name	RI Exp. ^1^	RI Lit. ^2^	*Thuja* *occidentalis*	*Cupressus* *sempervirens*	*Juniperus* *communis*	*Picea* *mariana*	*Pinus* *sylvestris*	*Pinus* *pinaster*
				Area (%) ^3^					
1	β-Picoline	880	871	- ^4^	-	-	-	0.05	0.06
2	Santolinatriene	899	902	-	0.05	-	-	0.04	-
3	*cis*-4-Heptenal	903	903	-	-	-	-	0.03	-
4	Isocitronellene	918	919	-	0.03	-	-	0.01	-
5	Tricyclene	921	926	-	0.21	-	-	1.38	0.12
6	α-Thujene	930	929	2.91	0.92	-	-	0.10	0.02
7	α-Pinene	932	937	5.43	45.06	0.90	15.73	37.18	53.25
8	β-Citronellene	943	948	-	6.06	-	-	0.01	-
9	α-Fenchene	945	949	-	0.33	-	-	0.08	-
10	Thuja-2,4(10)-diene	952	953	-	0.37	-	-	-	-
11	Camphene	958	952	4.99	1.53	0.03	18.66	7.84	1.51
12	*trans*-Pinane	967	971	-	0.04	-	0.02	-	-
13	Sabinene	970	974	17.09	0.03	8.27	-	-	0.02
14	*meta*-Cymene	971	976	-	0.06	0.49	-	0.10	0.14
15	β-Pinene	972	979	1.17	6.73	-	3.47	0.94	11.02
16	*trans*-Isolimonene	975	984	-	-	-	0.02	-	0.08
17	*trans-para*-Menthane	980	985	-	-	-	0.03	-	-
18	3-Octanone	985	986	-	-	0.04	-	-	-
19	Neobergamate	986	987	-	-	-	0.10	-	-
20	*cis*-Pinane	988	988	-	0.04	-	-	-	-
21	β-Myrcene	990	991	1.25	1.09	1.83	2.58	0.10	2.80
22	δ-2-Carene	1001	1000	-	0.04	-	-	0.04	-
23	*para*-Mentha-1(7),8-diene	1004	1003	-	-	-	-	-	0.16
24	α-Phellandrene	1005	1007	-	0.09	0.41	0.33	0.06	-
25	δ-3-Carene	1011	1009	-	25.02	-	6.63	37.52	12.17
26	1,4-Cineole	1015	1016	-	0.27	-	0.07	0.03	0.03
27	α-Terpinene	1023	1017	1.33	0.15	8.50	0.09	0.02	0.03
28	*ortho*-Cymene	1018	1024	-	-	-	-	0.03	-
29	*para*-Menth-1-ene	1025	1023	-	-	-	0.03	-	-
30	*para*-Cymene	1030	1025	4.03	0.11	1.94	0.68	0.37	1.23
31	Limonene	1034	1030	4.99	6.94	4.80	21.51	0.63	9.38
32	Eucalyptol	1032	1031	-	0.20	-	-	0.21	-
33	β-Phellandrene	1038	1032	-	-	-	0.01	-	-
34	*trans*-β-Ocimene	1048	1046	-	-	0.05	-	-	-
35	γ-Terpinene	1058	1058	2.61	0.07	12.18	0.09	0.03	0.06
36	*cis*-Sabinene hydrate	1071	1069	-	-	4.41	-	-	-
37	Terpinolene	1085	1086	-	4.26	3.40	0.76	0.03	0.30
38	Fenchone	1089	1089	16.81	-	-	-	-	-
39	*trans*-Sabinene hydrate	1104	1099	-	-	17.74	-	-	-
40	α-Pinene oxide	1098	1101	-	0.02	-	-	0.22	0.19
41	Linalool	1100	1102	-	0.03	-	0.05	-	-
42	β-Thujone	1113	1114	11.09	-	-	-	-	-
43	*endo*-Fenchol	1116	1119	-	0.04	-	-	-	-
44	Fenchyl alcohol	1117	1123	-	-	-	-	-	0.09
45	*cis*-Limonene oxide	1131	1134	-	-	-	-	0.10	0.04
46	*cis*-*para*-Mentha-2,8-dien-1-ol	1135	1138	-	0.02	1.69	-	0.03	0.17
47	*trans*-*para*-Menth-2-en-1ol	1136	1139	-	-	0.97	-	0.16	-
48	*trans*-Pinocarveol	1138	1140	-	-	-	-	0.03	0.11
49	*trans* -Myroxide	1142	1141	-	0.16	-	-	-	0.09
50	*trans*-Verbenol	1143	1145	-	-	-	-	0.18	0.11
51	Camphor	1151	1149	-	-	-	0.07	-	-
52	Camphene hydrate	1155	1156	-	-	-	0.06	-	0.02
53	Borneol	1172	1173	-	-	0.05	1.28	5.33	0.11
54	Isoneral	1174	1174	-	-	-	-	0.07	-
55	Terpinen-4-ol	1183	1184	3.51	-	20.06	0.07	-	0.04
56	*trans*-Isocarveol	1187	1188	-	-	-	-	0.04	0.05
57	para-Cymen-8-ol	1190	1189	-	-	0.06	-	-	-
58	α-Terpineol	1195	1195	-	-	4.01	0.23	0.06	0.73
59	Isopulegol	1196	1196	-	-	-	-	0.09	-
60	*cis*-Piperitol	1200	1198	-	-	0.50	-	-	-
61	γ-Terpineol	1201	1200	-	-	0.03	-	0.05	-
62	Carveol	1202	1206	-	-	-	-	-	-
63	Verbenone	1205	1208	-	-	-	-	0.02	-
64	*trans*-Piperitol	1213	1209	-	-	0.52	-	-	-
65	Fenchyl acetate	1225	1219	-	-	-	0.85	-	-
66	Nerol	1227	1229	-	-	0.04	-	-	-
67	*trans*-Chrysanthenyl acetate	1231	1231	-	-	0.02	-	-	-
68	Car-3-en-2-one	1245	1249	-	-		-	0.04	-
69	Linalyl acetate	1258	1250	-	-	2.52	0.08	-	-
70	*trans*-Ascaridol glycol	1275	1270	-	-	0.12	-	-	-
71	Bornyl acetate	1286	1285	9.25	-	-	25.19	0.02	1.33
72	*trans*-Sabinyl acetate	1288	1289	13.54	-	-	-	-	-
73	Terpin-1-en-4-yl acetate	1305	1296	-	-	0.04	-	-	-
74	γ-Elemene	1344	1335	-	-	0.04	-	-	-
75	α-Cubebene	1346	1348	-	-	-	-	-	0.05
76	α-Terpinyl acetate	1347	1349	-	-	-	0.37	-	-
77	α-Longipinene	1349	1352	-	-	-	-	-	0.07
78	*cis*-Geranyl acetate	1367	1361	-	-	0.02	-	-	-
79	α-Copaene	1375	1375	-	-		-	-	0.11
80	*trans*-Geranyl acetate	1386	1380	-	-	0.03	-	-	-
81	α-Gurjunene	1411	1406	-	-	-	0.04	-	-
82	Longifolene	1407	1412	-	-	-	-	-	0.82
83	*trans*-Caryophyllene	1424	1429	-	-	2.74	0.59	0.04	1.80
84	α-Humulene	1464	1454	-	-	0.13	0.30	-	0.17
85	Bicyclogermacrene	1507	1497	-	-	1.23	-	-	-
86	β-Bisabolene	1515	1508	-	-	-	0.02	-	-
87	δ-Cadinene	1517	1518	-	-	-	-	-	0.09
88	epi-Longipinanol	1548	1558	-	-	-	-	0.02	-
89	Spathulenol	1589	1576	-	-	0.06	-	-	1.35
90	Caryophyllene oxide	1595	1587	-	-	0.09	-	6.70	-
91	Humulene epoxide II	1613	1613	-	-	-	-	-	0.08
92	*allo*-Aromandendrene epoxide	1649	1644	-	-	0.05	-	-	-

^1^ Experimentally calculated linear retention index. ^2^ Literature-derived retention index according to the NIST 23 Mass Spectral Library. ^3^ Calculated by peak area normalization. ^4^ Not detected.

**Table 2:** Effects of essential oils (EOs) on selected rumen microorganisms, including inhibitory activity (MIC, IC_50_) and growth stimulation.

Essential Oils	*Butyrovibrio* *fibrisolvens*				*Prevotella albensis*				*Lactobacillus delbrueckii* ssp. *lactis*				*Streptococcus bovis*			
	MIC ^1^ ppm	IC_50_ ^2^ ppm	GS ^3^		MIC ppm	IC_50_ ppm	GS		MIC ppm	IC_50_ ppm	GS		MIC ppm	IC_50_ ppm	GS	
*Thuja* *occidentalis*	800	400	25	2×	800	400	-	-	1600	800	100	4.5×	1600	400	100	1.5×
*Juniperus* *communis*	800	200	-	-	800	25	-	-	1600	400	-	-	1600	800	-	-
*Pinus* *sylvestris*	800	400	50	1.4×	800	200	-	-	6400	1600	-	-	1600	800	-	-
*Pinus pinaster*	200	100	-	-	100	50	-	-	800	400	-	-	6400	3200	-	-
*Cupressus* *sempervirens*	3200	800	-	-	1600	800	-	-	1600	400	-	-	3200	1600	-	-
*Picea mariana*	800	100	-	-	800	200	-	-	1600	800	-	-	1600	800	-	-

^1^ MIC (minimum inhibitory concentration). ^2^ IC_50_ (half maximal inhibitory concentration). ^3^ GS, growth stimulation, is expressed as fold increase relative to control. Results are presented as means of three culture tubes at each concentration.

**Table 3:** Effects of *Pinus sylvestris* essential oils on in vitro fermentation characteristics.

Time	CON	*Pinus sylvestris*		SEM	*p*-Value
		25 ppm	50 ppm		
**pH**					
4 h	6.73	6.68	6.72	0.02	0.32
24 h	6.61	6.62	6.63	0.03	0.82
**NH_3_-N, mg L^−1^**					
4 h	265.56	262.12	286.72	8.45	0.68
24 h	283.73	283.40	308.00	11.20	0.15
**NH_3_, mg L^−1^**					
4 h	206.76	209.56	216.67	13.10	0.12
24 h	220.27	220.10	239.00	12.65	0.23
**NH_4_^+^, mg L^−1^**					
4 h	213.56	228.76	264.67	10.85	0.42
24 h	257.87	267.60	291.02	11.42	0.31
**Total VFA, mg mL^−1^**					
4 h	1.19	1.04	0.83	0.07	0.06
24 h	1.09	1.15	1.04	0.08	0.07
**Individual VFA, mg mL^−1^**					
**Acetate**					
4 h	0.678	0.661	0.483	0.04	0.04
24 h	0.587	0.690	0.640	0.03	0.12
**Propionate**					
4 h	0.295	0.202	0.179	0.02	0.01
24 h	0.299	0.238	0.207	0.02	0.11
**Butyrate**					
4 h	0.217	0.177	0.169	0.01	0.07
24 h	0.212	0.217	0.191	0.01	0.42
**Acetate to propionate ratio**					
4 h	2.298	3.272	2.698	0.03	0.05
24 h	1.963	2.899	3.092	0.04	0.04
**Methane, mL L^−1^**					
4 h	2.41	2.71	4.97	0.31	<0.01
24 h	18.48	16.54	23.58	1.42	0.05

**Table 4:** Ammonia (NH_3_), NH_3_-N, NH_4_^+^, volatile fatty acids (VFA), and ruminal pH in cows supplemented with *Pinus sylvestris* essential oil.

Parameter	Group	Period of Treatment				SEM	*p*-Value		
		0 d	3 d	7 d	14 d		PG	PT	PG × T
pH	CON	6.85	6.97	6.62	6.70	0.04	0.45	<0.01	0.13
	*P. sylvestris*	6.78	6.96	6.59	6.58	0.05			
NH_3_-N, mg L^−1^	CON	191.00	113.50	308.00	197.50	14.12	0.589	<0.01	0.31
	*P. sylvestris*	143.00	92.75	292.50	198.00	12.30			
NH_3_, mg L^−1^	CON	243.00	138.00	374.50	235.00	16.73	0.554	<0.01	0.40
	*P. sylvestris*	181.00	113.00	355.50	240.75	14.8			
NH_4_^+^, mg L^−1^	CON	235.00	146.00	396.50	255.00	18.4	0.61	<0.01	0.28
	*P. sylvestris*	177.25	119.50	376.75	255.00	15.2			
Total VFA, mg mL^−1^	CON	2.92	2.10	2.70	3.19	0.27	0.36	0.07	0.23
	*P. sylvestris*	3.19	2.27	1.12	3.93	0.18			
**Individual VFA, mg mL^−1^**									
Acetate	CON	1.87	1.37	1.42	2.05	0.12	0.32	0.02	0.01
	*P. sylvestris*	2.03	1.48	0.66	2.54	0.15			
Propionate	CON	0.65	0.45	0.52	0.71	0.04	0.22	0.01	0.07
	*P. sylvestris*	0.69	0.49	0.26	0.86	0.06			
Butyrate	CON	0.40	0.299	0.33	0.43	0.03	0.48	0.02	0.09
	*P. sylvestris*	0.46	0.30	0.20	0.53	0.04			
A:P ratio	CON	2.88	3.04	2.73	2.89	0.24	0.24	0.01	0.08
	*P. sylvestris*	2.94	3.02	2.54	2.95	0.29			
Methane, ppm	CON	7705.79	7397.27	15,135.99	21,264.11	1245.31	0.01	<0.01	0.08
	*P. sylvestris*	7498.68	8214.95	14,383.41	15,146.17	1180.22			

*p*-Value: PG for the effect of group, T for the effect of time, and PG × T for the interaction between group and time.

**Table 5:** Mean values of lipid and biochemical parameters in blood of cows.

Parameter	Group	Treatment Period			SEM	*p*-Value		
		0 d	7 d	14 d		PG	PT	PG × T
Glucose mmol L^−1^	CON	3.81	3.37 ^a,1^	3.47	0.11	0.08	0.04	0.03
	*Pinus sylvestris*	3.78	3.53 ^b^	3.60	0.23			
BHBA ^2^ mmol L^−1^	CON	0.28	0.24	0.22	0.04	0.15	0.08	0.12
	*Pinus sylvestris*	0.25	0.26	0.28	0.09			
NEFA ^3^ mmol L^−1^	CON	0.21	0.29	0.22	0.05	0.42	0.31	0.28
	*Pinus sylvestris*	0.20	0.20	0.23	0.12			
Chol. ^4^ mmol L^−1^	CON	2.35	2.71	2.37	0.28	0.34	0.18	0.11
	*Pinus sylvestris*	2.23	2.63	2.52	2.32			
HDL ^5^ mmol L^−1^	CON	1.54	1.47	1.43	0.12	0.28	0.15	0.09
	*Pinus sylvestris*	1.37	1.56	1.59	0.19			
LDL ^6^ mmol L^−1^	CON	0.27	0.40	0.31	0.06	0.45	0.22	0.35
	*Pinus sylvestris*	0.25	0.32	0.27	0.17			
TG ^7^ mmol L^−1^	CON	0.21	0.21	0.20	0.04	0.55	0.48	0.62
	*Pinus sylvestris*	0.19	0.19	0.18	0.09			
TP ^8^ g L^−1^	CON	73.13	74.00	74.75	4.21	0.05	0.52	0.18
	*Pinus sylvestris*	80.03	79.43	82.28	5.62			
Alb. ^9^ g L^−1^	CON	30.65	26.75 ^A^	29.90	1.42	0.15	0.04	0.01
	*Pinus sylvestris*	29.33	30.58 ^B^	29.15	2.23			
AST ^10^ U L^−1^	CON	49.93	63.90	53.09	3.12	0.22	0.18	0.09
	*Pinus sylvestris*	48.13	53.56	53.13	4.13			
ALT ^11^ U L^−1^	CON	22.59	20.69	19.79	2.05	0.48	0.61	0.58
	*Pinus sylvestris*	19.46	21.24	20.79	3.07			
GGT ^12^ U L^−1^	CON	22.61	28.14	22.93	2.12	0.65	0.12	0.15
	*Pinus sylvestris*	22.84	25.28	25.00	2.56			
TAS ^13^ mmol L^−1^	CON	1.15	1.04	1.14	0.08	0.41	0.35	0.18
	*Pinus sylvestris*	1.13	1.11	1.15	0.12			

^1^ Different letters within a column indicate significant differences between groups (^a,b^—*p* < 0.05; ^A,B^—*p* < 0.01); ^2^ beta-hydroxybutyrate; ^3^ non-esterified fatty acids; ^4^ cholesterol; ^5^ high-density lipoprotein; ^6^ low-density lipoprotein; ^7^ triglycerides; ^8^ total protein; ^9^ albumin; ^10^ aspartate aminotransferase; ^11^ alanine aminotransferase; ^12^ gamma-glutamyl transferase; ^13^ total antioxidant status; *p*-Value: PG for the effect of group, T for the effect of time, and PG × T for the interaction between Group and Time.

## Data Availability

The raw data supporting the conclusions of this article will be made available by the authors on request.

## Supplementary Materials

The following supporting information can be downloaded at: https://www.mdpi.com/article/10.3390/molecules31101769/s1, Table S1: Significant differences in the relative abundance of selected microbial families. The table summarizes bacterial families showing statistically significant differences (*p* < 0.05) in relative abundance between experimental groups and/or sampling timepoints in the rumen microbiota. Values are presented as mean ± SD; Table S2: Detailed per-sample alpha diversity indices; Table S3: Alpha diversity.
